# A High-Sensitivity Method Based on Advanced Optical Waveguide Technology to Detect HBsAg

**DOI:** 10.1155/2021/8719147

**Published:** 2021-07-26

**Authors:** Pingping Xiao, Xiaoxiong Hu

**Affiliations:** ^1^Department of Electronic Information Engineering, College of Physics and Engineering Technology, Yichun University, Yichun, Jiangxi 33600, China; ^2^Department of Infectious Diseases, The People's Hospital of Yichun City, Yichun, Jiangxi 33600, China

## Abstract

A novel method for the detection of the hepatitis B surface antigen (HBsAg) at low concentrations, using the ultrahigh-order guided mode acting as the probe excited by a symmetrical metal-cladding waveguide, is proposed. The method using the fact of the minimum value of the absorption peaks is proportional to the concentration of the sample to be detected to realize the detection of the hepatitis B virus at extremely low concentrations. It is realized that the low concentration of the HBsAg measurement relied on the principle of the minimum value of the absorption peak and the concentration having a good linear relationship. The measurement results indicate that this new method can precisely detect HBsAg at the concentrations in the lower region of the clinical gray area (i.e., below 20 ng/mL), the lower region of the current clinical gray area of HBsAg (below 20 ng/ml) can be measured, and the resolution can be reached (2 ng/mL).

## 1. Introduction

It is well known that the infection of hepatitis B virus (HBV) can cause acute and chronic hepatitis B and even can lead to death of the person who got infected [1]. Patients with HBV for a long time may progress to liver fibrosis, cirrhosis, liver cancer, and a series of complications such as portal hypertension syndrome and hepatic encephalopathy, which seriously threaten their lives [2]. According to the incomplete statistics, all around the world, there are about two billion people infected with HBV in their lives [3, 4], and about 12% of them have developed chronic hepatitis B virus infections. And there are about 650,000 people who lost their lives because of liver failure, cirrhosis, and liver cancer caused by HBV infection [5–7]. The rate of the cirrhosis and hepatocellular carcinoma (HCC) caused by HBV infection is 30% and 45%, respectively [8], and therefore, it is very significant to develop methods that are able to precisely detect the concentrations of HBV in human blood, for both clinical medicine and our society.

Qualitative detection of HBsAg is the main basis for hepatitis B patients in clinics. The common detection methods of HBsAg include colloidal gold immunochromatography assay (GICA) [9], enzyme-linked immunosorbent assay (ELISA), chemiluminescence method (CLIA) [10], and electrochemiluminescence immunoassay (ECLIA) [11]. There are advantages and disadvantages for the four main detection methods. At present, the most widely used technology is the ELISA because of its high sensitivity in the world. It is very simple, low cost, and sensitive for the GICA. CLIA can be used to detect lower concentration or microconcentration samples in the reaction, but the detection is limited. ECLIA is a new label immunoassay technology after radioimmunoassay, enzyme immunoassay, and chemiluminescence immunoassay. The detection conditions are monopolized, the detection equipment is expensive, and the clinical accessibility is poor.

To improve the resolution of detection, an optical detection technology, surface plasmon resonance (SPR) technology, is proposed by some researchers to detect the concentrations of the matter contained in liquid samples. The SPR technology uses the resonance absorbance peak as the probe excited at the interface of metal and nonmetal by the interaction between laser and free electrons in metal and has high detection sensitivities. The SPR technology also has the following outstanding advantages: no need of bulky equipment, real-time detection, label-free measurement, and little sample amount requirement [12]. Due to the outstanding advantages of real-time detection and little sample amount requirement, the SPR technology has been widely used in the studies of interactions between biomolecules and the combination dynamics between antibodies and antigens [13]. Biacore is the most typical commercial instrument that uses the SPR technology.

In our preliminary studies, we found that although the SPR technology already has wide applications, its operation principle has some issues or concerns. Firstly, the surface plasma wave can only be transmitted at the interface between the metal and medium, and then the full width at half maximum (FWHM) of the attenuation of the total reflection absorption peak is large because metals absorb light in the range of visible and near-infrared wavelengths. Secondly, since the coupling angle of the prism coupling system of the surface plasma wave is large, the prism coupling system has a large effective refractive index. These two issues severely limited the further improvement on the detection sensitivity of the SPR sensor. Additionally, since the SPR technology uses the evanescent field as the probe, the probing range is only within a few hundred nanometers, which limits the applications of the SPR technology only to the detection of macromolecular samples. Although several new technologies, such as leaky optical waveguide (LW) [14], reverse symmetry waveguide (RSW) [15], and long-range surface plasma wave (LSPR) [16], have been developed, these newly developed technologies made no breakthroughs in the operation principle of the SPR technology, and like in the existing SPR technology, in these newly developed technologies, the sample is still placed in the evanescent field where the light field is weak. With the consideration of these issues in the operation principle of the existing SPR technology, this paper proposes a new high-sensitivity detection technology to detect HBsAg based on the symmetrical metal-cladding waveguide technology.

## 2. Operation Principle

### 2.1. Detection Principle

**Figure 1:**
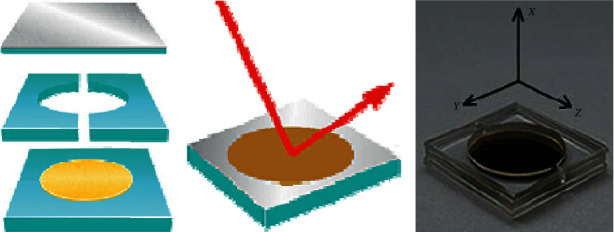
Schematic diagram to illustrate the structure of the symmetrical metal-cladding waveguide.

A schematic diagram for the structure of the sensor chip named symmetrical metal-cladding waveguide (SMCW) is illustrated in Figure 1. From bottom to top, the sensor chip consists of the substrate glass (0.5 mm), the thick metal (gold or silver) film (200 nm) sputtered on one side, the gasket glass (0.5 mm) with a circular hole in the center, the upper glass (0.5 mm), and the thin metal (gold or silver) film (30 nm) sputtered on one side.

**Figure 2:**
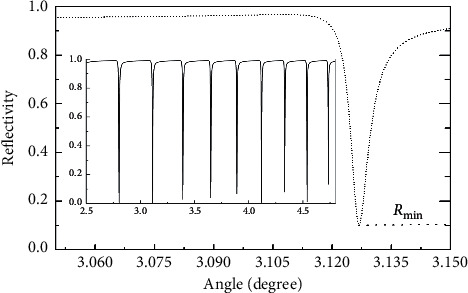
Absorption peaks of the attenuated total reflection.

The attenuated total reflection spectrum shown in Figure 2 can be obtained when the incident angle of the incident beam scans from 0 to 90° in the three-layer symmetrical metal-cladding waveguide structure. Figure 2 shows one of the absorption peaks of the attenuated total reflection spectrum at a small incident angle.

According to Cao [17], the minimum of the absorption peak of the attenuation total reflection in the structure of a three-layer metal-cladding waveguide can be written as

(1) Rmin∝1−4ImΔβLImβ0+ImΔβL/Imβ02,

where Im(*β*^0^) is the intrinsic loss determined by the absorption of the waveguide material and Im(Δ*β*^*L*^) is the radiation loss determined by the thickness of the upper metal film.

From equation (1), obviously, when Im(Δ*β*^0^) = Im(Δ*β*^*L*^), i.e., when the intrinsic loss is equal to the radiation loss, the minimum value of the attenuated total reflection peak is equal to zero. When the sensor chip for the experiments is prepared, the structural parameters of the sensor chip are determined, and then the radiation loss of the sensor chip is also determined. If there is a change in the concentration of the ingredient to be detected in the sample, the intrinsic loss will vary, and then the minimum of the absorption peak of the attenuation total reflection will also change. According to this principle, the detection of sample concentration by measuring the minimum of the absorption peak of the attenuation total reflection may be realized.

### 2.2. Sensitivity Analysis

**Figure 3:**
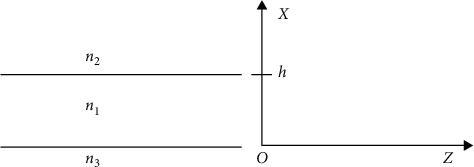
Schematic diagram of the three-layer waveguide.

The basic structure of a three-layer planar optical waveguide is shown in Figure 3.

The TE mode distribution of the electric field in a three-layer planar optical waveguide can be obtained as

(2) $$\begin{array}{c} E_{y}\left(x\right)=\cos\left(\right)\left\{\begin{array}{cc} A\;\exp\left(\alpha_{0}x\right), & -\infty <x<0,\\ \frac{A\;\cos\left(\kappa_{1}x-\phi_{13}\right)}{\cos\;\phi_{13}}, & 0<x<h,\\ \frac{A\;\cos\left(\kappa_{1}h-\phi_{13}\right)\exp\left[-\alpha_{2}\left(x-h\right)\right]}{\cos\;\phi_{13}}, & h<x<+\infty , \end{array}\right. \end{array}$$

where *ϕ*_12_ = tan^−1^(*α*_2_/*κ*_1_), *ϕ*_12_ = tan^−1^(*α*_3_/*κ*_1_), $\alpha_{3}=\left(k_{0}/\sqrt{N_{\text{eff}}^{2}-n_{3}^{2}}\right)$, $\alpha_{2}=\left(k_{0}/\sqrt{N_{\text{eff}}^{2}-n_{2}^{2}}\right)$, and $k_{1}=\left(k_{0}/\sqrt{n_{1}^{2}-N_{\text{eff}}^{2}}\right)$.

The dispersion equation of the TE guided mode can be easily derived from the boundary conditions as follows [18]:

(3) $$\begin{array}{c} \kappa_{1}h=m\pi +\arctan\left(\frac{\alpha_{3}}{\kappa_{1}}\right)+\arctan\left(\frac{\alpha_{2}}{\kappa_{1}}\right),\;m=0,1,2,3,\ldots . \end{array}$$

By differentiating both sides of equation (3), we can have

(4) $$\begin{array}{c} \frac{\Delta N_{\text{eff}}}{\Delta n_{1}}=\frac{\left(n_{1}/N_{\text{eff}}\right)\cdot \left[h+\left(\alpha_{3}/\left(\kappa_{1}^{2}+\alpha_{3}^{2}\right)\right)+\left(\alpha_{2}/\left(\kappa_{1}^{2}+\alpha_{2}^{2}\right)\right)\right]}{h+\left(1/\alpha_{3}\right)+\left(1/\alpha_{2}\right)}. \end{array}$$

The power *P*_*ab*_ that the TE mode carries (from *a* to *b*) in the waveguide is obtained from

(5) $$\begin{array}{c} P_{ab}=\frac{\beta }{2\omega \mu_{0}}\cdot \int_{a}^{b}{\left[E_{y}\left(x\right)\right]}^{2}\text{d}x, \end{array}$$

where *μ*_0_ is the vacuum permeability. The region from *a* to *b* can be on either the substrate or the core layer of the waveguide:

(6) $$\begin{array}{c} \frac{P_{1}}{P_{\text{Total}}}=\frac{\left[h+\left(\alpha_{3}/\left(\kappa_{1}^{2}+\alpha_{3}^{2}\right)\right)+\left(\alpha_{2}/\left(\kappa_{1}^{2}+\alpha_{2}^{2}\right)\right)\right]}{h+\left(1/\alpha_{3}\right)+\left(1/\alpha_{2}\right)}, \end{array}$$

where *P*_1_ is the optical power coupled into the sample and *P*_Total_ is the total power input by the laser beam. The derivative of the effective refractive index of the guided mode can be obtained as

(7) $$\begin{array}{c} \frac{\text{d}N_{\text{eff}}}{\text{d}n_{1}}=\frac{n_{1}}{N_{\text{eff}}}\cdot \frac{P_{1}}{P_{\text{Total}}}, \end{array}$$

where *n*_1_ is the refractive index of the sample to be detected and *N*_eff_ is the effective refractive index of the resonant mode.

Similarly, the derivative of the effective refractive index of the TM mode can be obtained as

(8) $$\begin{array}{c} \frac{\text{d}N_{\text{eff}}}{\text{d}n_{1}}=\frac{n_{1}}{N_{\text{eff}}}\cdot \frac{P_{1}}{P_{\text{Total}}}\cdot \frac{1-\delta }{h_{eg}}, \end{array}$$

where *h*_*eg*_ = *h* + (*n*_3_^2^*n*_1_^2^*α*_3_/(*n*_0_^4^*κ*_1_^2^ + *n*_1_^4^*α*_3_^2^)) + (*n*_1_^2^*n*_2_^2^*α*_2_/(*n*_2_^4^*κ*_1_^2^ + *n*_1_^4^*α*_2_^2^)).

Then, the uniform sensitivity formula *S* of an optical waveguide sensor with the resonant mode can be written as

(9) $$\begin{array}{c} S=\frac{\text{d}N_{\text{eff}}}{\text{d}n_{1}}=\eta \cdot \frac{n_{1}}{N_{\text{eff}}}\cdot \frac{P_{1}}{P_{\text{Total}}}, \end{array}$$

where *η* is a physical quantity related to the polarization of the laser and structure of the sensor, i.e., *η* = 1 for the TE mode and *η* = ((1 − *δ*)/*h*_*eg*_) for the TM mode.

Since (*n*_*i*_/*N*_eff_) ≪ 1 and (*P*_*i*_/*P*_Total_) ≪ 1 for the surface plasmon resonance structure, it can be seen from equation (9) that *S* ≪ 1. However, for a sensor based on the three-layer symmetrical metal-cladding waveguide structure, since the analyte is located in the region of the oscillating field where the light energy is very high, the incident laser light is almost all coupled into the waveguide, i.e., *P*_*i*_ ≈ *P*_Total_. Since the incident angle *θ*_air_ is very small, the effective refractive index *N*_eff_ = *κ*_0_*n*_air_sin  *θ*_air_ approaches to zero. According to equation (9), in principle, the sensitivity *S* approaches to infinity, and therefore, the sensitivity of this detection method based on the three-layer symmetrical metal-cladding waveguide structure is very high.

**Figure 4:**
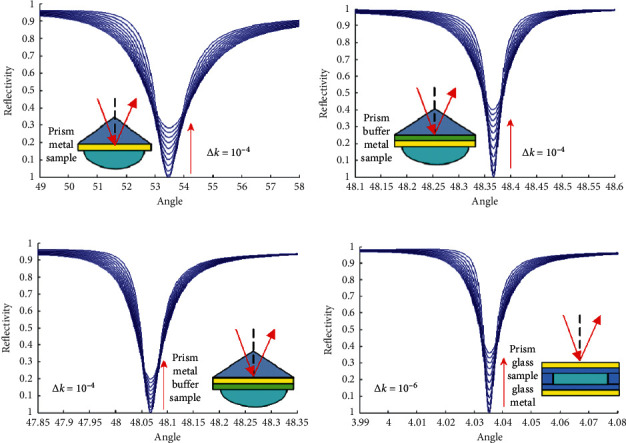
Variations of the ATR with the extinction coefficient for the four different sensing technologies. (a) SPR. (b) LSPR. (c) RSW. (d) SMCW.

Simulations for the sensitivities of the SPR sensing technology using the prism coupled technology, the LSPR sensing technology using the prism coupled technology, and the RSW sensing technology using prism coupled technology and SMCW sensing technology with free-space coupling technology were conducted. The parameters used in the simulations and the results of these simulations for the four sensing technologies are listed in Table 1 and shown in Figure 4, respectively. The simulation results indicate that the sensitivity of the sensing technology proposed here using the variation of the minimum value of the absorption peak (*R*_min_) with the extinction coefficient of the sample and based on the three-layer symmetrical metal-cladding waveguide structure is at least two to three orders of magnitude higher than those of the other three traditional optical sensing technologies.

## 3. Sample Preparation

The original, national, and quantitative standard sample of HBsAg (NIBSC code: 00/880) was provided by the World Health Organization. The content of HBsAg in each bottle of freeze-dried serum is 16 ng, and the concentration is 160 ng/mL after adding 0.1 mL sterile ultrapure deionized water into each bottle. According to the dilution factor of the sample solution, a total of seven standard experimental samples with different concentrations besides pure distilled water were prepared.

Further sample preparation process is as follows:

1. Add 100 *μ*L of each of the seven standard experimental samples with different concentrations of HBsAg, respectively, to the seven same wells coated with the hepatitis B surface antibody, shake well, and then place the wells in an incubator of 37°C for about 1 hour.
2. Add 50 *μ*L of biotin-labeled capture antibody (secondary antibody) to each well, and then place the wells in an incubator of 37°C for about 30 minutes.
3. Use detergent to wash several times to wash away the unbound capture antibody, and then add 50 *μ*L of horseradish peroxidase-labeled streptavidin to each well, shake well, and place the wells in an incubator of 37°C for 30 minutes.
4. After washing, add 100 *μ*L of the reaction substrate o-phenylenediamine (OPD) to each well, shake well, and then place the wells in an incubator of 37°C for 30 minutes.
5. Terminate the reaction in each well by adding 100 *μ*L of acid, and then six orange-red liquids of different shades of the color after the oxidation by OPD are obtained. The used experimental samples of various specified concentrations and their corresponding labels are listed in Table 2.

## 4. Experimental Measurement

### 4.1. Setup

**Figure 5:**
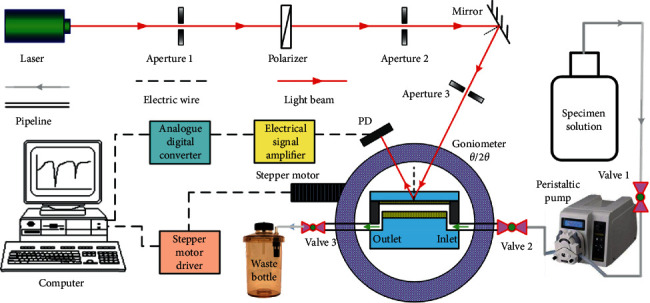
Schematic diagram of the experimental setup used for the measurements.

The schematic diagram of the experimental setup used for the measurements is shown in Figure 5. The model number of the laser source used was MW-SL-532 whose output laser has a center wavelength of 532 nm and has a maximum output optical power of about 30 mW. The two small holes (aperture 1 and aperture 2) were to ensure the collimation of the laser line. The polarization controller (polarizer) was used to select the TM polarization state of the laser. The high-reflection mirror was used to change the direction of the optical path to ensure that the incident light from the laser hits the center spot of the sensor chip. The sensor chip was placed on the goniometer which was connected to the two independently rotating parts controlled by a stepping motor, the inside part and the outside part, and the angular velocity of the outside part was twice of that of the inside part.

The photodetector (PD) used was a photoelectrical detection tube manufactured by ALPHALAS Inc. in Germany (model no. UPD-35-UVI), with a response wavelength range of 350–1700 nm. In the photoelectrical detection tube, the response signal is amplified by an analog signal amplifier, and then the amplified analog signal is converted into a digital signal by an analog digital signal conversion circuit. The final digital signal is input into the computer signal acquisition module, the acquired digital signals are further processed by self-programmed computer software, and then the measurement results will display on its monitor. In the experimental setup, the injection of the specimens into the sensor chip was realized by an electric peristaltic sampler with a low noise.

### 4.2. Measurement

At the beginning of the measurement, the inner rotating part that held the sensor chip was manually adjusted so that the incident point of the laser was at the center spot of the sensor chip, and the incident angle of the laser was about 2°. The incident angle of the laser was controlled by the computer program to scan in the range between 2° and 60°. The narrowest half-height full width and the lowest ATR absorption peak on the computer screen was selected to be the working peak. Then, the starting and ending laser incident angles of the selected working peak were set as the incident angle scanning range of the laser for the measurements, and then the laser was scanned back and forth within the set incident angle scanning range multiple times to make sure this scanning can repeat well. Since the gap between the forward and backward screws will induce errors in the measured experimental data, the laser was scanned in the same one direction to acquire the experimental data.

After all the moving parts are ensured in their stable working conditions, sample A was slowly injected into the sample chamber of the sensor chip by using an electric peristaltic pump. After the solution of the sample fully filled the sample chamber of the sensor chip, the peristaltic pump was shut off, and the solution of the sample in the sample chamber was let to stabilize for several minutes before the measurement started. Computer software was then turned on to control the laser to scan in one direction from the set starting to the set ending incident angles. The absorption peak acquired during each scan was stored as the measurement data. To obtain more reliable experimental data, during the measurements, the components in the experimental setup were maintained stationary, and the laser scanning was made sure to be just in one direction.

The sensor chip must be rinsed with sterile ultrapure deionized water multiple times before it is used to measure the next sample until all the seven samples are measured, and the measurement process for each of the samples was a repeat of the above process to measure sample A.

## 5. Results and Discussion

### 5.1. Results

**Figure 6:**
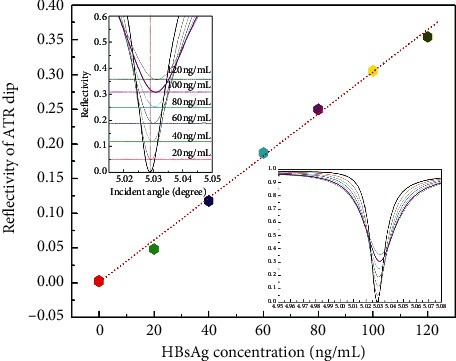
Variation of the minimum reflectivity related to different applied HBsAg concentrations.

Figure 6 shows the plot of the minimum value of the absorption peak (*R*_min_) versus the concentration of the HBsAg standard sample. The lower right inset in Figure 6 shows all the measured curves of the attenuating total reflection absorption peak, and the upper left inset in Figure 6 is an enlarged view of the bottom peak portion of the measured curves shown in the lower right inset in Figure 6. The plot showed a very good linear relation between measured *R*_min_ and the concentration of HBsAg in the range of the sample concentrations measured. It was also noticed that the minimum value of the ATR absorption peak varied about 0.05 with a variation of 20 ng/mL in the concentration of the HBsAg sample. Due to the resolutions of the data acquisition and computer display, the resolution of this measurement method to differentiate the minimum value (*R*_min_) of the ATR peak was not less than 0.004. Considering the influences of other noises in the measurement process, the sensitivity of this optical waveguide detection method will not be lower than 2 ng/mL.

### 5.2. Discussions

The plot shown in Figure 6 indicates that the minimum value of the ATR absorption peak (*R*_min_) is not absolutely equal to zero when the concentration of HBsAg is zero. The reason for this observation is that Im(*β*^0^) and Im(Δ*β*^*L*^) of the optical waveguide are not absolutely equal since the thickness of the upper coupling metal film layer is not absolutely equal to the optimum thickness at the time of the preparation of the sensor chip, but this will not have any impact on the measurement accuracy of this detection method.

We noticed that, in Figure 6, the attenuating total reflection absorption peaks of different samples were not only shifted with each other in the vertical direction but also slightly shifted in the horizontal direction. These horizontal and vertical shifts of the attenuating total reflection absorption peaks were caused by the real and imaginary parts of the refractive index of the sample, and then the horizontal and vertical shifts of the attenuating total reflection absorption peaks were totally not related. Therefore, the horizontal shifts of the attenuating total reflection absorption peaks will also not have any influence on the picked measurement parameter *R*_min_ of the proposed detection method.

We also noticed that, in Figure 6, there were some deviations of the experimental data from the theoretical predictions. We believe these deviations of the experimental data from the theoretical predictions were mainly related to the following three factors:

1. When the sensor chip was prepared, there existed island structures on the surface of the upper metal film, and then the uniformity of the thickness of the upper metal film was not guaranteed.
2. The parallelism and half-width of the output laser of the selected laser model exceeded the theoretical expectations.
3. The operation temperature of the sensor chip increased when irradiated by the laser, which caused an inconsistent temperature environment for the sensor chip before and during the experimental measurement. We believe that this issue can be solved if the working device with the sensor chip can be placed in a high-precision incubator.

## 6. Conclusions

Theoretical and experimental results indicate that the lowest resolution of the sensing technology introduced here, based on the symmetrical metal-cladding waveguide configuration, to detect HBsAg at low concentrations can reach 2 ng/mL, which will be useful in the diagnosis and treatment of hepatitis B patients. According to the detection principle of this novel method, this method can be used to detect the quantitative level of any substance as long as the substance can be colored with chromogenic agents, such as hepatitis B e antigen (HBeAg) and C-reactive protein (CRP). The application of optical detection technologies may provide a new technical route for clinical detections.

## Figures and Tables

**Table 1:** Parameters used in the simulations for the four sensing technologies in the resonant mode.

Sensor	SPR	LSPR	RSW	SMCW
Prism (*ε*_0_)	3.24	3.24	3.24	1.0
Buffer layer (*ε*_1_, *d*_1_)		1.78, 1060 nm		1.0
Upper metal film (*ε*_2_, *d*_2_)	−12 + *i*0.5, 50 nm	−12 + *i*0.5, 15 nm	−12 + *i*0.5, 50 nm	−12 + *i*0.5, 39 nm
Upper glass (*ε*_3_, *d*_3_)				2.25, 0.5 mm
Guide wave film (*ε*_4_, *d*_4_)			2.25, 1500 nm	1.78, 0.5 mm (sample)
Bottom glass (*ε*_5_, *d*_5_)				2.25, 0.5 mm
Bottom metal film (*ε*_6_, *d*_6_)				−12 + *i*0.5, 200 nm
Sample (*ε*_7_)	1.78	1.78	1.78	
Δ*k*	10^−4^	10^−4^	10^−4^	10^−6^

Laser wavelength: *λ* = 488 nm; Δ*k*: the change of the imaginary part of the refractive index of the sample.

**Table 2:** The specimens used in the experimental measurements with various concentrations of HBsAg.

Label of the specimen	Concentration (ng/mL)
A	0
B	20
C	40
D	60
E	80
F	100
G	120

## Data Availability

The data used to support the findings of this study are available from the corresponding author upon reasonable request and with the permission of funders.
